# Immunomodulatory Effects of Bone Marrow-Derived Mesenchymal Stem Cells in a Swine Hemi-Facial Allotransplantation Model

**DOI:** 10.1371/journal.pone.0035459

**Published:** 2012-04-25

**Authors:** Yur-Ren Kuo, Chien-Chang Chen, Shigeru Goto, Yu-Ting Huang, Chun-Ting Wang, Chia-Chun Tsai, Chao-Long Chen

**Affiliations:** 1 Center for Composite Tissue Allotransplantation, Kaohsiung Chang Gung Memorial Hospital and Chang Gung University College of Medicine, Kaohsiung, Taiwan; 2 Department of Plastic and Reconstructive Surgery, Kaohsiung Chang Gung Memorial Hospital and Chang Gung University College of Medicine, Kaohsiung, Taiwan; 3 Liver Transplantation Program and Department of Surgery, Kaohsiung Chang Gung Memorial Hospital and Chang Gung University College of Medicine, Kaohsiung, Taiwan; University of Southern California, United States of America

## Abstract

**Background:**

In this study, we investigated whether the infusion of bone marrow-derived mesenchymal stem cells (MSCs), combined with transient immunosuppressant treatment, could suppress allograft rejection and modulate T-cell regulation in a swine orthotopic hemi-facial composite tissue allotransplantation (CTA) model.

**Methodology/Principal Findings:**

Outbred miniature swine underwent hemi-facial allotransplantation (day 0). Group-I (n = 5) consisted of untreated control animals. Group-II (n = 3) animals received MSCs alone (given on days −1, +1, +3, +7, +14, and +21). Group-III (n = 3) animals received CsA (days 0 to +28). Group-IV (n = 5) animals received CsA (days 0 to +28) and MSCs (days −1, +1, +3, +7, +14, and +21). The transplanted face tissue was observed daily for signs of rejection. Biopsies of donor tissues and recipient blood sample were obtained at specified predetermined times (per 2 weeks post-transplant) or at the time of clinically evident rejection. Our results indicated that the MSC-CsA group had significantly prolonged allograft survival compared to the other groups (*P*<0.001). Histological examination of the MSC-CsA group displayed the lowest degree of rejection in alloskin and lymphoid gland tissues. TNF-α expression in circulating blood revealed significant suppression in the MSC and MSC-CsA treatment groups, as compared to that in controls. IHC staining showed CD45 and IL-6 expression were significantly decreased in MSC-CsA treatment groups compared to controls. The number of CD4+/CD25+ regulatory T-cells and IL-10 expressions in the circulating blood significantly increased in the MSC-CsA group compared to the other groups. IHC staining of alloskin tissue biopsies revealed a significant increase in the numbers of foxp3^+^T-cells and TGF-β1 positive cells in the MSC-CsA group compared to the other groups.

**Conclusions:**

These results demonstrate that MSCs significantly prolong hemifacial CTA survival. Our data indicate the MSCs did not only suppress inflammation and acute rejection of CTA, but also modulate T-cell regulation and related cytokines expression.

## Introduction

Mesenchymal stem cells (MSCs) from the bone marrow are multi-potential non-hematopoietic progenitor cells in the adult marrow capable of differentiating into various mesenchymal cell types. Previous studies have revealed that MSCs do not express immunogenic co-stimulatory molecules, such as B7-1, B7-2, or CD40 [1]–[2]. Therefore, it is likely that they are unable to stimulate alloreactive T-cells. The immunomodulatory effects of MSCs have been demonstrated both *in vitro* and *in vivo* [3]. Studies have indicated that donor MSCs are potent inhibitors of T-cell proliferation in mixed lymphocyte cultures, thus preventing graft-versus-host disease (GVHD) caused by bone marrow transplantation (BMT) and prolonging skin allograft survival in rodent models [4].

**Figure 1:**
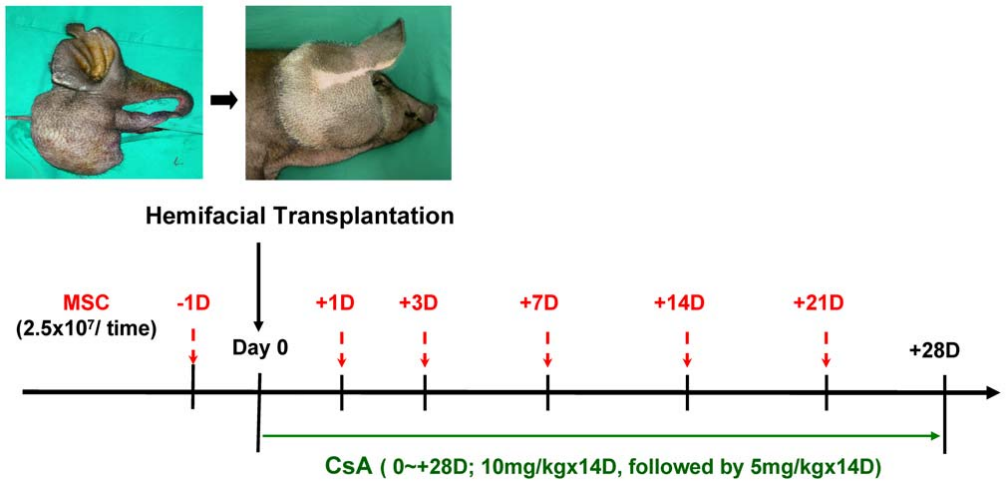
The flowchart of the timetable of this study is shown. Outbred miniature swine underwent hemi-facial allotransplantation (day 0). Group I consisted of the control cohort that did not undergo immunosuppressive therapy. Group II received MSCs alone (2.5×10^7^ MSCs/dose, given on days −1, +1, +3, +7, +14, and +21). Group III received cyclosporine A (CsA) for 4 weeks (days 0 to +28; 10 mg/kg for 2 weeks, followed by 5 mg/kg for 2 weeks). Group IV received CsA (days 0 to +28) and MSCs (given on days −1, +1, +3, +7, +14, and +21).

In a previous study, we clearly showed that donor bone marrow-derived MSC therapy, in addition to BMT, after total body irradiation and short-term cyclosporine A (CsA) treatment significantly improved allotransplant survival without signs of GVHD in a swine heterotopic hind-limb composite tissue allotransplantation (CTA) model [5]. We further demonstrated that the administration of multiple donor MSCs without BMT has similar results on allotransplant survival in the same model [6]. Our results led us to speculate that BMT is unnecessary to prolong CTA survival if MSCs are used as an immunosuppressant [5]. The miniature swine hemi-facial CTA model (consisting of skin paddle, muscle, ear cartilage, and lymphoid gland tissue) had been established by our institute recently [7]. The difference between the hemifacial CTA model and the hind-limb model is that this hemi-facial model does not contain donor vascularized bone, but it does include more alloskin area and lymphoid gland tissue. However, there have not been any studies that have assessed the effects of MSCs in a large animal facial allotransplant study. To re-confirm this hypothesis and to test another CTA model for pre-clinical study, we designed the current protocol to investigate whether multiple treatments with MSCs combined with treatment with a transient immunosuppressant yielded reproducible results and prolonged allotransplant survival in a miniature swine hemi-facial model (Fig. 1). We also examined how the immunoregulatory effects of MSCs could contribute to prolonged CTA survival.

## Results

### MSC therapy combined with transient immunosuppressant treatment prolongs hemi-facial allotransplant survival

**Figure 2:**
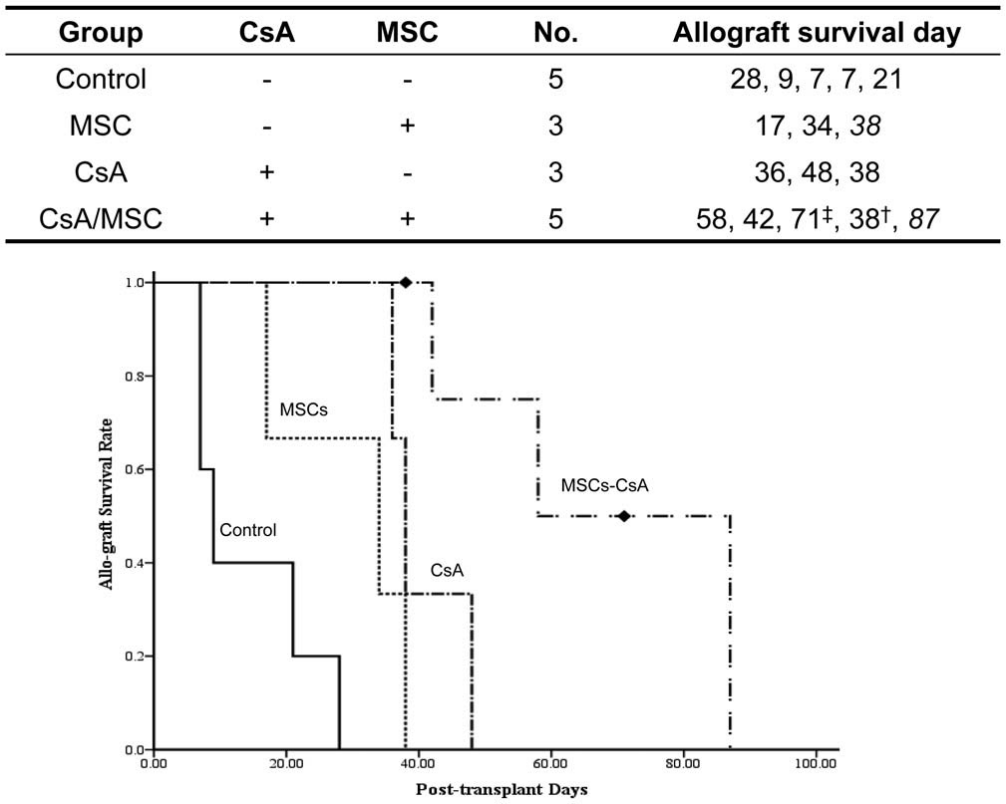
Treatment with MSCs and transient immunosuppressive therapy prolongs hemi-facial allotransplant survival. Multiple MSC injections in the absence of immunosuppression (group II) produced a trend but not a statistically significant increase in allotransplant survival compared to controls. Allotransplants in group III animals, which received short-term CsA treatment, displayed delayed rejection and survival for 36–48 days post-transplant (p = 0.018). The combination of MSC injections with CsA treatment (group IV) significantly prolonged allotransplant survival compared to other groups (p<0.001). One swine was sacrificed (day 38 post-transplantation) due to severe infection, although the allotransplant was still viable. Another died incidentally during biopsy (day 71 post-transplantation).

Allograft recipients treated with multiple short-term MSC injections in the absence of immunosuppressant treatment (group II) revealed a trend but no statistically significant increase in allograft survival 17–38 days post-transplantation compared to the controls, which survived for 7–28 days (p = 0.123). Allotransplantation, along with short-term CsA treatment, for 4 weeks in group III resulted in delayed rejection compared to that of controls (p = 0.018). However, treatment with MSCs in addition to transient CsA treatment (group IV) resulted in significant increases in allograft survival when compared to other experimental groups (Fig. 2, p<0.05).

### MSC therapy with short-term immunosuppressant treatment suppresses hemi-facial allotransplant rejection

**Figure 3:**
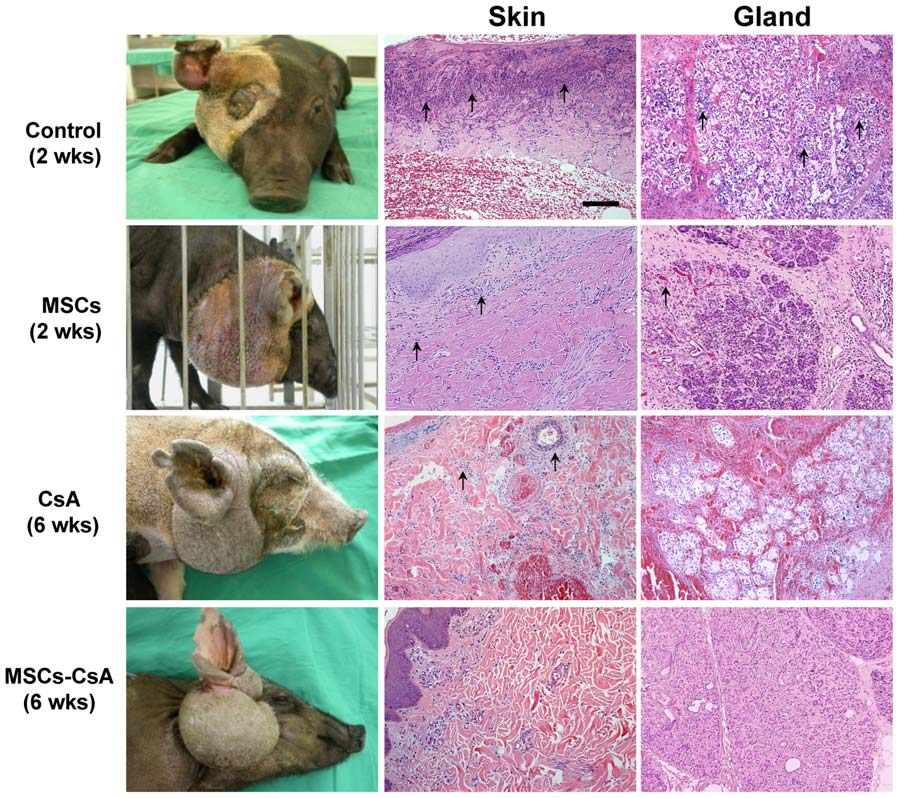
Histopathological examination of MSCs and short-term cyclosporine A (CsA) decreases allotransplant rejection. Histopathological evaluation revealed signs of severe rejection (grade III) including diffuse, dense polymorphic inflammatory cells infiltrate beneath the dermal-epidermal junction in grafted skin and lymphoid gland tissues of untreated controls at 2 weeks post-transplantation (arrow). Animals in the MSC alone group showed moderate rejection (grade II) with a slighted dense dermal infiltrate inflammatory cells around dermal blood vessels and diffusing interstitially in lymphoid gland tissues at 2 weeks post-transplantation. Animals in the CsA group showed a dense perivascular dermal lymphocytic infiltrate, extending focally into the epidermis (grade II–III) at 6 weeks post-transplantation. Animals in the MSC-CsA group revealed small number perivascular lymphocytes infiltrated in the alloskin (grade I) or lymphoid gland tissue (grade I) when compared to other groups at 6 weeks post-transplantation. Photo magnification is 100× in skin and lymphoid gland tissue. Scale bar = 10 µm.

Histopathological evaluation revealed severe graft acute rejection (grade III), including inflammatory cell infiltrates in grafted skin and in lymphoid gland samples of untreated controls at 2 weeks post-transplantation. Allograft biopsies from animals treated with MSCs alone revealed moderate to severe rejection (grades II–III) in the grafted skin in the dermal-epidermal junction, subcutaneous tissue, and gland tissue at 2 weeks post-transplantation. CsA-treated animals revealed grade I rejection in the alloskin and gland tissue at 2 weeks post-transplant, while there was mild to moderate lymphocyte infiltration in the gland tissue and alloskin (grade II–III) at 6 weeks post-transplantation. However, the MSC-CsA group revealed mild lymphocyte infiltration in the grafted skin (grade I) or lymphoid gland tissue (grade I) when compared to other groups at 2 weeks and 6 weeks post-transplantation (Fig. 3). This indicated MSCs could modulate early allograft acute rejection.

### MSC therapy increased regulatory T-cell populations in the peripheral blood and allotransplant tissues

**Figure 4:**
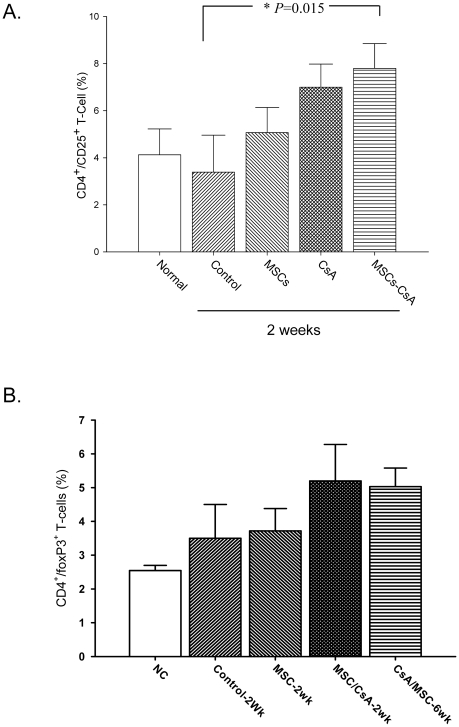
Treatment with MSCs and short-term CsA increases regulatory T-cell populations in recipient peripheral blood samples. Flow cytometry analysis indicated that CD4+/CD25+ regulatory T-cell populations were significantly increased in animals treated with MSCs and CsA compared to controls **(Fig. 4A**, *P = 0.015). In contrast, flow cytometry analysis indicated that CD4+/foxp3+ regulatory T-cell populations were a trend increased in animals treated with MSCs and CsA compared to controls (**Fig. 4B**).

**Figure 5:**
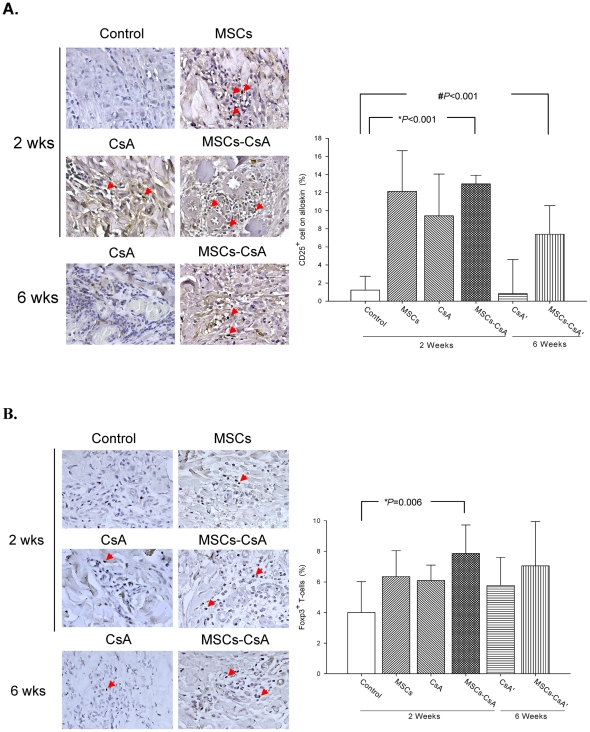
Treatment with MSCs and a transient immunosuppressant increased CD25+ and Foxp3+ T-cell populations in transplanted alloskin tissues. IHC staining of biopsy tissue in donor skin from the MSC-CsA group revealed significantly increased numbers of CD25+ T-cells in the subcutaneous and dermis layers compared to the control group (**Fig. 5A**, *P<0.05). The MSC-CsA group revealed significantly increased numbers of foxp3+ T-cells in the subcutaneous and dermis layers compared to control groups 2 weeks post-transplantation (**Fig. 5B**, *P<0.05). Photo magnification is 400×. Scale bar = 50 µm.

Flow cytometric analysis of recipient peripheral blood revealed that the proportion of CD4+/CD25+ T-cells increased significantly in animals treated with MSCs-CsA at 2 weeks post-transplantation compared to controls (Fig. 4A). The CD4+/foxp3+ expressions in circulating blood have a tendency to increase in MSC-CsA group as compared with those in the other groups (Fig. 4B). These indicated MSC and CsA regulate T-cells expression in the early stage post-transplant. In contrast, IHC staining of alloskin tissue biopsies revealed a significant increase in the numbers of CD25+ and foxp3+ T-cells in the subcutaneous and dermis layers of animals treated with MSCs-CsA compared to the other groups at 2 weeks post-transplantation (Fig. 5A and Fig. 5B). The CD25+ T cells were expressed on similar site of foxp3+ cells. The levels of CD25+ and foxp3+ T-cells in the MSC-CsA group were still higher at 6 weeks post-transplantation compared to other groups.

### MSC and transient immunosuppressive therapy regulated the pro-inflammatory and anti-inflammatory cytokines

**Figure 6:**
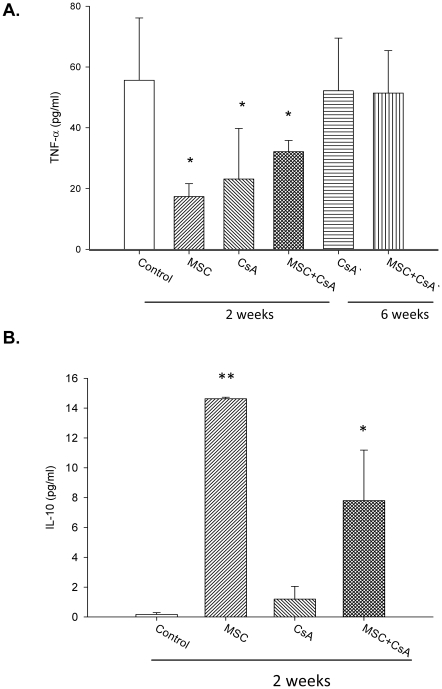
MSC and transient immunosuppressive therapy regulated the pro-inflammatory and anti-inflammatory cytokines. **T**he concentrations of the soluble forms of TNF-α determined by ELISA revealed a significant decrease in animals treated with MSCs and MSC-CsA groups at 2 weeks post-transplantation compared to controls (Fig. 6A, * *P*<0.05). The concentrations of the soluble forms of IL-10 revealed that IL-10 level was statistically increased in animals treated with MSC and MSC-CsA groups at 2 weeks post-transplantation compared to controls (**Fig. 6B**, * *P*<0.01).

The concentrations of the soluble forms of TNF-α, IL-10, and TGF-βl were determined by ELISA after the various treatments. Analysis of recipient peripheral blood serum revealed the TNF-α level had a significant decrease in animals treated with MSCs and MSC-CsA groups at 2 weeks post-transplantation, as compared to those in controls (Fig. 6A). The IL-10 level had a statistically significant increase in animals treated with MSC and MSC-CsA groups at 2 weeks post-transplantation, as compared to controls (Fig. 6B). In contrast, TGF-βl levels of recipient peripheral blood serum showed a trend of increase but no significant difference between MSC groups and the controls.

**Figure 7:**
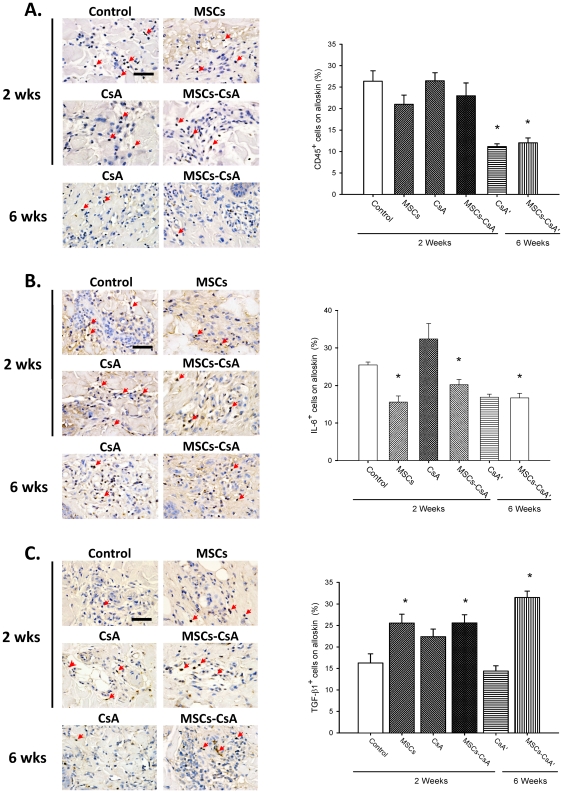
The IHC staining of alloskin biopsy revealed significantly lower numbers of CD45 and IL-6 positive cells in the subcutaneous and dermis layers of skin from animals treated with MSC and MSC-CsA groups, compared to the controls (Fig. 7A and 7B, *P<0.05). The IHC staining of alloskin biopsy revealed significantly increased the numbers of TGF-βl positive cells in the subcutaneous and dermis layers of skin from animals treated with MSC and MSC-CsA groups at 6 weeks post-transplantation compared to controls (Fig. 7C * ***P*** **<0.05**). Scale bar = 50 µm.

The IHC staining of alloskin biopsy revealed significantly lower numbers of CD45 and IL-6 positive cells in the subcutaneous and dermis layers of skin from animals treated with MSC and MSC-CsA groups, compared to the controls (Fig. 7A and 7B). The alloskin biopsy revealed significantly increased the numbers of TGF-βl positive cells in the subcutaneous and dermis layers of skin from animals treated with MSC at 2 weeks post-transplantation and MSC-CsA groups at 2 and 6 weeks post-transplantation compared to controls (Fig. 7C).

## Discussion

Composite tissue allotransplantations are not routinely performed for tissue reconstruction because of the potentially harmful adverse effects associated with lifelong administration of immunosuppressive agents, which is required for highly antigenic tissue. Therefore, the development of novel and non-toxic strategies that circumvent the long-term use of immunosuppressants is critical.

Recently, the immunoregulatory properties of MSCs were described. Studies indicated MSC infusion had good effects on many immuno-diseases [3], [8]–[10]. Our previous study revealed that the infusion of donor bone marrow-derived MSCs could prolong allotransplant survival; in addition, we showed that the presence of MSCs correlated with increases in regulatory T-cell populations in a swine heterotopic hind-limb model (consisting of skin, muscle, knee joint, vascularized bone and bone marrow) [9]–[11]. In the present study, we investigated whether multiple treatments with MSCs combined with a transient immunosuppressant could reproduce the increase in allograft survival using a miniature swine hemi-facial allotransplant model. However, the different animal models of CTA may cause the different outcome by MSC infusion. Our present study showed that MSCs alone without short-term CsA had just a tendency to prolong the survival but not statistically prolonged swine hemifacial CTA survival. These discrepancies may be explained by the difference of the amount of alloskin between swine hemifacial transplant model and other CTA models. Especially, swine hemifacial CTA model owns more alloskin and lymphoid gland tissue without donor vascularized bone marrow. As far as we know, allo-skin is the highest antigenicity to induce immune rejection. Skin transplantation with donor bone marrow might provide more stem cells to modulate recipient T-cell regulation.

In contrast, the recipients treated with short-term CsA were characterized by a significant increase in allotransplant survival compared to the untreated controls. Interestingly, the recipients treated with MSCs combined with a transient CsA regimen displayed more significant prolongation of allograft survival than the CsA alone group, although the allograft was eventually rejected. Although animal number in each group may not be sufficient as a preclinical study, however, the number is minimally enough for statistical analysis [6]. Results indicated the MSC/CsA group statistically prolonged allotransplant survival as compared to that in controls. Multiple MSC infusions in combination with conventional CsA treatment would indeed constitute a novel strategy to substantially prolong hemi-facial composite tissue allotransplant survival. Therefore, adjustment of the dosage or the timing of MSCs infusion in hemi-facial CTA are still needed to assess the function of MSCs.

Allogeneic skin and lymphoid gland tissue are considered to be the most antigenic tissue involved in hemi-facial CTA procedures [11]–[12]. In our hemi-facial model, histopathological analysis of untreated controls revealed substantial, severe acute rejection in alloskin and lymphoid gland tissue (grade III) 2 weeks post-transplantation. Although the MSCs alone group did not display a statistically significant prolongation of allotransplant survival, donor skin and lymphoid gland biopsies revealed less rejection signs (grade II–III) than controls 2 weeks post-transplantation. This indicates that the MSCs, at least in part, suppress allograft rejection. The recipients that received a therapy that combined MSC infusions with short-term CsA treatment did not demonstrate apparent signs of rejection in either grafted skin or lymphoid gland biopsies at 6 weeks post-transplantation. In contrast, the TNF-α level determined by ELISA revealed a significant decrease in animals treated with MSCs and MSC/CsA groups at 2 weeks post-transplantation compared to controls. The IHC staining of alloskin biopsy revealed significantly lower numbers of CD45 and IL-6 positive cells in the subcutaneous and dermis layers of skin from animals treated with MSC and MSC/CsA groups, as compared to those in controls. These results indicate that multiple MSC infusions combined with short-term immunosuppressant therapy could prevent acute rejection and prolong CTA survival.

Recently, the immunomodulatory properties of MSC infusions have been described. Studies indicated that MSCs modulate immune responses through the induction of regulatory T-cells [2], [13]–[15]. Additional studies have suggested that MSCs may inhibit T-cell activation, thereby prolonging skin graft survival in a rodent model [8]. Studies indicated MSCs have immuno-modulatory properties and involve regulatory T-cells while cyclosporine did not increase the percentage of CD4+/CD25+/foxp3+ T-cells in a rat cardiac allograft [16]–[17]. In this study, single MSCs or CsA alone did not have a significant effect to affect the percentage of CD4+/CD25+ and CD4+/foxp3+ regulatory T-cells. However, FACS analysis of the CD4+/CD25+ and CD4+/foxp3+ regulatory T-cell populations in recipient peripheral blood revealed significantly expanded regulatory T-cell populations in animals treated with MSCs and CsA compared to controls at 2 weeks post-transplantation. Immunohistochemical staining of allograft tissue showed significant increases in CD25+ and Foxp3+ T-cell populations in the subcutaneous and dermis layers of the skin. The reason should be the synergistic effect of MSC injections and CsA administration increase allotransplant survival and induce T-cell regulation. In contrast, our study demonstrated the concentrations of IL-10 in recipient peripheral blood serum revealed statistical increase in animals treated with MSC and MSC-CsA groups at 2 weeks post-transplantation compared to controls. The IHC staining of alloskin biopsy revealed significantly lower numbers of TGF-βl positive cells in the subcutaneous and dermis layers of skin from animals treated with MSC and MSC/CsA groups at 6 weeks post-transplantation compared to controls. This demonstrated the possible mechanism of MSC and CsA upregulation of Treg cells is mediated immunomodulatory effect and induced a more anti-inflammatory effect.

In summary, this pre-clinical study indicates that multiple infusions of donor MSCs combined with transient immunosuppressant treatment can effectively prolong allotransplant survival in a swine hemi-facial allotransplant model. This large animal CTA model reconfirms the hypothesis that MSCs have immunomodulatory effects mediated by the suppression of allograft rejection and induction of T-cell regulation, suggesting that MSC infusion is a novel strategy for increasing CTA survival.

## Materials and Methods

### Animals

Twenty-four outbred domestic miniature swine (Lan-Yu strain and Hwa-Ban strain; age, 3 months; weight, 12–20 kg) were included in this study. The miniature swine is an indigenous breed from Lau-Yu Islet, southeast of Taiwan. This study was conducted in accordance with the Guide for the Care and Use of Laboratory Animals published by the National Institutes of Health, U.S.A. Experiments were conducted using the Institutional Animal Care and Use Committee (IACUC) protocol approved by the Kaohsiung Chang Gung Memorial Hospital, Taiwan.

### Hemi-facial composite tissue allotransplant swine model

Orthotopic hemi-facial allotransplantation (Hwa-Ban strain to Lan-Yu strain) was performed as described previously [7]. The swine hemi-facial transplantation tissue consisted of ear cartilage, auricular nerve, parotid gland, lymphoid tissue, and muscle with surrounding hemi-facial skin paddle [7]. The vascular territories of the composite allotransplant supplied by the superficial temporal artery and its branches originating from the carotid artery were defined. The common carotid artery and external jugular vein were dissected as the vascular pedicle of the allotransplant. After flushed heparinized-saline solution, the hemi-facial flap was secured and sutured in the recipient. End to end venous anastomosis was performed between the external jugular vein of the donor and recipient. Then, end-to-side anastomosis between the common carotid artery of the recipient and donor was performed under operating microscope magnification.

### Experimental design

Miniature swine underwent orthotopic hemi-facial allotransplantation. Group I (n = 5) was the control cohort, and the animals did not undergo immunosuppressive therapy. Group II (n = 3) animals received MSCs alone (2.5×10^7^ MSCs/dose, given on days −1, +1, +3, +7, +14, and +21). Group III (n = 3) animals received cyclosporine A (CsA) for 4 weeks (days 0 to +28; 10 mg/kg for 2 weeks, followed by 5 mg/kg for 2 weeks). Group IV (n = 5) animals received CsA (same protocol as group III; days 0 to +28) and MSCs (2.5×10^7^ MSCs/dose, given on days −1, +1, +3, +7, +14, and +21). The flowchart of the timetable in this study is shown in **Fig. 1**. This work was supported in part by Chang Gung Research Project (Contract No. grant CMRPG-850081 and CMRPG-860452). Swine viability and signs of allograft rejection were continuously monitored postoperatively. The clinical experimental endpoint was defined as desquamation and necrosis of the entire area of donor skin.

### Culturing of MSCs

Bone marrow cells from donors were harvested and isolated 2 weeks prior to CTA using methods described previously [5]. Briefly, bone marrow cells were suspended in low-glucose Dulbecco's Minimal Essential Medium (DMEM), 10% fetal bovine serum (FBS), antibiotic/antimycotic, and glutamax, and the cells were plated in 6-well dishes. The cultures were incubated at 37°C in a 5% CO2 atmosphere. After 4 hrs of subtraction and removal of adherent cells, the non-adherent cells were transferred to 25-T subculture flasks. When they were 70–80% confluent, adherent cells were trypsinized (0.05% trypsin at 37°C for 5 min), harvested, and expanded into 75-T flasks. MSCs were expanded in culture and demonstrated positive surface staining for CD44, CD90, MHC class I, and CD106 but not for CD45, MHC class II, and CD80/B7-1, as revealed by flow cytometry [5]. MSCs were tested for their ability to differentiate into adipocytes, osteoblasts, and chondrocytes before use [5].

### Histological evaluation of graft rejection

Biopsies of donor skin, gland lymphoid tissue and cartilage were obtained at specified predetermined times or at the time of clinically evident rejection [7], [18]. According to the severity of pathological changes, rejection grades using the Banff classification were applied [19].

### Flow cytometric assessment of T-cell regulation

Flow cytometric analysis was performed on peripheral blood samples of recipients collected on specified days post-transplant. Blood sample was incubated in the dark (room temperature) with mouse anti-porcine CD25-fluorescein isothiocyanate (FITC) (Pharmigen, USA) and mouse anti-pig CD4-phycoerythrin (PE). Another sample was stained with a combination of FITC-conjugated mouse anti-rat Forkhead box P3 (foxp3) antibody (eBioscience) and CD4-labeled PE. After incubation, red blood cells were lysed, and the remaining cells were centrifuged. The cells were then analyzed by flow cytometry (FACScan, Becton Dickinson)

### Cytokine detection of TNF-α, IL-10, and TGF-β1

The soluble forms of tumor necrosis factor-alpha (TNF-α), interleukin-10 (IL-10), and transforming growth factor-βl (TGF-βl) were determined by an enzyme-linked immunosorbent assay (ELISA) kit (R&D system, Minneapolis, MN). Blood serum samples were collected at predetermined specific days post-transplantation.

### Immunohistochemical (IHC) staining

Tissue sections were subjected to IHC for CD25, foxp3, and TGF-β1 to investigate T-cell regulation, CD45 and IL-6 for pro-inflammatory reaction. A horseradish peroxidase-diaminobenzidine (HRP–DAB) kit was used (BioGeneX, USA). After endogenous peroxidase activity was blocked with 3% hydrogen peroxide, tissue sections were stained with mouse anti-porcine CD25 (Serotec, UK), anti-foxp3 (Serotec, UK), anti-TGF-β1 (Abcam, UK), and anti-CD45 antibody (Serotec, UK). The reaction sections were incubated with a biotinylated anti-mouse antibody as a secondary antibody. Visualization of specific binding was developed by enzymatic conversion of the chromogenic substrate 3, 3′-DAB into a brown precipitate by HRP. After counterstaining with hematoxylin, donor tissue sections were mounted, cleared, and coverslipped [20].

### Histomorphometric analysis

For immunostaining quantification, the tissue sections were analyzed using a Zeiss Axioskop 2 Plus microscope (Carl Zeiss, Gottingen, Germany). Four randomly selected areas were then photographed at 400× magnification. All images were captured using a Cool CCD camera. The images were analyzed using the Image-Pro Plus image analysis software (Media Cybernetics, Silverbernetics Spring, MD) [20].

### Statistical analyses

Student's t-test or ANOVA was utilized to assess the statistical significance of differences among experimental groups. Log transformation prior to variance analysis and Student's t-test was performed to reconfirm normality and equal variances for flow cytometry data. Graft survival was compared among the different groups of transplanted animals using Kaplan-Meier analysis and the log-rank test. A P value of less than 0.05 was considered statistically significant.
